# Universality of interpersonal psychotherapy (IPT) problem areas in Thai depressed patients

**DOI:** 10.1186/1471-244X-10-87

**Published:** 2010-10-21

**Authors:** Peeraphon Lueboonthavatchai, Nuntika Thavichachart

**Affiliations:** 1 Department of Psychiatry, Faculty of Medicine, Chulalongkorn University, Bangkok, Thailand

## Abstract

**Background:**

Many studies have shown the efficacy of interpersonal psychotherapy (IPT) on depression; however, there are limited studies concerning the universality of the IPT problem areas in different countries. This study identifies whether the interpersonal problem areas defined in the IPT manual are endorsed by Thai depressed patients.

**Methods:**

The Thai Hamilton Rating Scale for Depression (Thai HRSD) and Thai Interpersonal Questionnaire were used to assess 90 depressed and 90 non-depressed subjects in King Chulalongkorn Memorial Hospital, during July 2007 - January 2008. The association between interpersonal problem areas/sociodemographic variables and depressive disorder were analyzed by chi-square test. A multivariable analysis was performed by using logistic regression to identify the remaining factors associated with depressive disorder.

**Results:**

Most of the subjects were young to middle-aged females living in Bangkok and the Central Provinces. All four interpersonal problem areas (grief, interpersonal role disputes, role transitions, and interpersonal deficits) were increased in the depressed subjects as compared to the non-depressed subjects, as were the sociodemographic variables (low education, unemployment, low income, and having a physical illness). Logistic regression showed that all interpersonal problem areas still remained problems associated with depression (grief: adjusted OR = 6.01, 95%CI = 1.93 - 18.69, p < 0.01; interpersonal role disputes: adjusted OR = 6.01, 95%CI = 2.18 - 16.52, p < 0.01; role transitions: adjusted OR = 26.30, 95%CI = 7.84 - 88.25, p < 0.01; and interpersonal deficits: adjusted OR = 2.92, 95%CI = 1.12 - 7.60, p < 0.05).

**Conclusion:**

All four interpersonal problem areas were applicable to Thai depressed patients.

## Background

Depressive disorder was one of the leading causes of worldwide disease burden, accounting for 4.46% of total disability-adjusted life-years (DALYs), and for 12.1% of total years lived with disabilities (YLDs) in 2000 [1]. Both major depressive disorder and dysthymic disorder are common depressive disorders, with a lifetime prevalence of about 15% and 3 - 6% respectively [2,3]. In Thailand, the lifetime prevalence of depressive disorder is about 5.7-20.9% [4]. Depressive disorder is believed to be caused by both biological and psychosocial factors.

Interpersonal psychotherapy (IPT), developed by Klerman and Weissman and based on the interpersonal theory of Adolf Meyer and Harry Stack Sullivan, has defined four interpersonal problem areas associated with the onset of a depressive episode [5-9]. The problem areas are: 1) grief or complicated bereavement, 2) interpersonal role disputes, 3) role transitions, and 4) interpersonal deficits [7-9]. IPT is thought to relieve depressive symptoms by helping patients resolve their interpersonal difficulties. IPT is a manualized form of psychotherapy and one of the evidence-based psychotherapies (EBTs) of depression [10-14]. Based on previous studies, IPT showed efficacy on treatment of depressive disorder and other psychiatric disorders [15-17]. However, there are still limited studies on the validity of interpersonal problem areas and whether they can be translated across cultures.

Previous studies focused on adverse life events related to depression. Holmes and Rahe reported that the most stressful life event was the death of a spouse [18]. Other important stressful life events included divorce, marital separation, detention in jail, death of a close family member, and major injury or illness [18]. Kendler reported that the stressful life events predicting the onset of major depression included death of a close relative, assault, serious marital problems, and divorce or breakup (odds ratio of more than 10) [19]. Markowitz found a correlation between interpersonal problem improvement and reduction of depressive symptoms in 24 patients [20,21].

Interpersonal difficulties, such as grief, interpersonal conflicts, life transitions, and social isolation seem to be universal human experiences; however, they may differ between cultures due to different socio-cultural experience. For example, Verdeli and Clougherty found that the fourth interpersonal problem area, interpersonal deficits, was not recognized as a problem area in Uganda because people in Uganda lived in tight-knit social groups and were never alone [22].

This study is aimed at identifying the interpersonal problem areas as defined in the IPT manual in Thai depressed patients. Studying interpersonal problems of Thai patients will help to determine whether these problems are present and an appropriate target of treatment, and will guide the adaptation of IPT for use in Thailand.

## Methods

Ninety depressed and ninety non-depressed subjects above 18 years old were recruited from the Department of Psychiatry, King Chulalongkorn Memorial Hospital in Bangkok during July 2007 - January 2008. Approval for the study was obtained from the Ethical Committee of the Institutional Review Board of the Faculty of Medicine, Chulalongkorn University. All 90 potential depressed subjects were consecutive patients who met the eligibility criteria during the period of study and were informed of the study's objectives and method. They voluntarily participated in the study and gave written informed consent. The inclusion criteria for the depressed subjects (cases) were that they were new cases (within 6 months) of major depressive disorder being diagnosed using the Diagnostic and Statistical Manual of Mental Disorders, 4^th ^edition, Text Revision (DSM-IV-TR) criteria [23], and that they had scores of at least 8 points on the Thai Hamilton Rating Scale for Depression (Thai HRSD) [24]. The exclusion criteria were schizophrenia and other psychotic disorders, bipolar disorders, organic mental disorders, substance use disorders, and mental retardation. The 90 non-depressed subjects or controls were recruited through the Department of Psychiatry and from family members or caregivers of psychiatric patients who were determined to have no depressive or other psychiatric disorders by psychiatric interview and had scores of less than 8 points on the Thai HRSD in the same period. All subjects completed two self-administered questionnaires: 1) the Demographic Data Form, and 2) the Thai Interpersonal Questionnaire.

The Thai HRSD is the Thai version of the Hamilton Rating Scale for Depression (HAM-D), the psychiatric rating scale widely used for evaluation of depressive disorder [25]. It was tested and found to have good validity and reliability in measuring the severity of depression in Thai depressed patients [24] (Cronbach's alpha coefficient = 0.74). The Thai HRSD is composed of 18 items and had a range of total scores from 0 to 57. The scores of 7 or under indicate an absence of depression; scores of 8 to 29 represent mild to major depression; and scores of 30 or above indicate severe depression or psychotic symptoms.

The Thai Interpersonal Questionnaire was developed for identifying interpersonal problem areas described in IPT and was adapted from the IPT manual [7]. The questionnaire is composed of four groups of items for identifying interpersonal problem areas: 1) grief or complicated bereavement (scores: 0 - 12), 2) interpersonal role disputes (0 - 15), 3) role transitions (0 - 9), and 4) interpersonal deficits (0 - 12). This questionnaire showed good validity and reliability (Cronbach's alpha coefficient for grief = 0.79; interpersonal role disputes = 0.96; role transitions = 0.96; and interpersonal deficits = 0.82). A high score on each subscale of an interpersonal problem area indicates a problem in adjusting in that area. The total range of scores for each problem area was divided into 3 intervals. The scores indicating the subjects' problem areas were the scores above the second interval that were compatible with the problem areas diagnosed by the clinical interview.

A statistical analysis was performed by using STATA for Windows version 8.0 software. The baseline demographic characteristics of the depressed (cases) and the non-depressed subjects (controls) were presented in number and percentage. The chi-square test was used to test the association between interpersonal problem areas/sociodemographic factors and depressive disorder. The strength of association between interpersonal problem areas/sociodemographic factors and depressive disorder was reported by using odds ratio (OR) with 95% confidence interval (95% CI). A multivariable analysis was performed by using logistic regression to identify the remaining factors associated with depressive disorder. A p-value of less than 0.05 was considered statistically significant.

## Results

One hundred eighty subjects participated in the study: 90 depressed and 90 non-depressed subjects (Table 1). Most of them were female (78.9%) and in the age range of 31 - 70 years (mean age = 42.8, SD = 12.0). About 62% were married, 33.3% were single, and 5% were separated, widowed, or divorced. About half had a bachelor's degree education or above. Nearly 70% of subjects were employed. Nearly half of the subjects had an income of 10,000 baht per month or above. Thirty-nine percent had at least one physical illness. Most (90%) lived in Bangkok and the Central Provinces (Table 1).

**Table 1:** Demographic characteristics of the depressed (n = 90) and the non-depressed (n = 90) subjects

Demographic characteristics	Depressed (n = 90) N, percent	Non-depressed (n = 90) N, percent	Total (n = 180) N, percent
**Gender**			
Female	71, 78.9%	71, 78.9%	142, 78.9%
Male	19, 21.1%	19, 21.1%	38, 21.1%
**Age**			
18 - 30 years	16, 17.8%	16, 17.8%	32, 17.8%
31 - 40 years	17, 18.9%	21, 23.3%	38, 21.1%
41 - 50 years	32, 35.6%	26, 28.9%	58, 32.2%
51 - 70 years	25, 27.8%	27, 30.0%	52, 28.9%
Mean ± SD	42.7 ± 11.9	43.0 ± 12.1	42.8 ± 12.0
Min, Max	18, 66	18, 68	18, 68
**Marital status**			
Couple	59, 65.6%	52, 57.8%	111, 61.7%
Others	31, 34.4%	38, 42.2%	69, 38.3%
**Educational level**			
Secondary school and lower	52, 57.8%	35, 38.9%	87, 48.3%
Bachelor's degree and higher	38, 42.2%	55, 61.1%	93, 51.7%
**Occupation**			
Employed	51, 56.7%	74, 82.2%	125, 69.4%
Unemployed	39, 43.3%	16, 17.8%	55, 30.6%
**Incomes (baht/month)**			
Lower than 10,000	56, 62.2%	38, 42.2%	94, 52.2%
10,000 and above	34, 37.8%	52, 57.8%	86, 47.8%
**Having a physical illness**			
Presence	43, 47.8%	28, 31.1%	71, 39.4%
Absence	47, 52.2%	62, 68.9%	109, 60.6%
**Residence**			
Bangkok and Central Provinces	78, 86.7%	85, 94.4%	163, 90.6%
Others	12, 13.3%	5, 5.6%	17, 9.4%

The scores on the Thai HRSD and Thai Interpersonal Questionnaire of the depressed and the non-depressed subjects are shown in Table 2. The scores of Thai HRSD, which indicate the severity of depression, varied from 0 - 43 (the depressed: 8 - 43 vs. the non-depressed: 0 - 7). The mean Thai HRSD score of total subjects was 14.32 (the depressed: 25.34 ± 8.58 vs. the non-depressed: 3.29 ± 2.67). The scores of all interpersonal problem areas in the depressed subjects were higher than the non-depressed subjects (Table 2).

**Table 2:** Scores on Thai HRSD and Thai Interpersonal Questionnaire of the depressed (n = 90) and the non-depressed (n = 90) subjects

Scores	Depressed (n = 90) Mean, SD	Non-depressed (n = 90) Mean, SD	Total (n = 180) Mean, SD
**Thai HRSD (0 - 52)**	25.34, 8.58	3.29, 2.67	14.32, 12.75
(Min, Max)	(8, 43)	(0, 7)	(0, 43)
**Thai Interpersonal Questionnaire**			
**Grief (0 - 12)**	2.87, 3.61	0.88, 1.53	1.87, 2.93
(Min, Max)	(0, 10)	(0, 6)	(0, 10)
**Interpersonal role disputes (0 - 15)**	7.61, 4.80	3.42, 4.14	5.52, 4.94
(Min, Max)	(0, 15)	(0, 14)	(0, 15)
**Role transitions (0 - 9)**	4.56, 3.19	0.54, 1.40	2.56, 3.18
(Min, Max)	(0, 9)	(0, 7)	(0, 9)
**Interpersonal deficits (0 - 12)**	4.20, 3.01	1.56, 1.97	2.68, 2.96
(Min, Max)	(0, 11)	(0, 7)	(0, 11)

The relationship between interpersonal problem areas/sociodemographic variables and depressive disorder is shown in Table 3. All interpersonal problem areas were associated with depressive disorder (grief: OR = 4.79, 95%CI = 2.14 - 11.29, p < 0.01; interpersonal role disputes: OR = 4.80, 95%CI = 2.42 - 9.56, p < 0.01; role transitions: OR = 31.00, 95%CI = 11.50 - 94.99, p < 0.01; and interpersonal deficits: OR = 7.42, 95%CI = 3.58 - 15.60, p < 0.01). In the problem area of role transitions, the common life changes that the subjects reported included separation and divorce, a move, job loss, health problems or physical illness, and financial problems. Among sociodemographic variables, the factors associated with depressive disorder included low education (OR = 2.15, 95%CI = 1.14 - 4.08, p < 0.05), unemployment (OR = 4.58, 95%CI = 2.32 - 9.11, p < 0.01), low income (OR = 2.25, 95%CI = 1.19 - 4.28, p < 0.05, and having a physical illness: OR = 2.03, 95%CI = 1.06 - 3.90, p < 0.05).

**Table 3:** Relationship between interpersonal problem areas/sociodemographic factors and depressive disorder in the depressed (n = 90) and the non-depressed (n = 90) subjects

Interpersonal problem areas and sociodemographic factors	Numbers (n = 180)			Odds ratio (OR)	95% CI Of OR	X^2^	p-value
	Depressed (90)	Non-depressed (90)	Total (180)				
**Interpersonal problem areas**							
							
**Grief**							
Exposed	36	11	47	4.79	2.14 - 11.29	18.00	< 0.0001**
Unexposed	54	79	133				
Total	90	90	180				
							
**Interpersonal role disputes**							
Exposed	67	34	101	4.80	2.42 - 9.56	24.57	< 0.0001**
Unexposed	23	56	79				
Total	90	90	180				
							
**Role transitions**							
Exposed	62	6	68	31.00	11.50 - 94.99	74.12	< 0.0001**
Unexposed	28	84	112				
Total	90	90	180				
							
**Interpersonal deficits**							
Exposed	57	17	74	7.42	3.58 - 15.60	36.72	< 0.0001**
Unexposed	33	73	106				
Total	90	90	180				
							
**Sociodemographic factors**							
							
**Educational level**							
Secondary school and lower	52	35	87	2.15	1.14 - 4.08	6.43	0.01*
Bachelor's degree and higher	38	55	93				
Total	90	90	180				
							
**Occupation**							
Unemployed	55	23	78	4.58	2.32 - 9.11	23.17	< 0.0001**
Employed	35	67	102				
Total	90	90	180				
							
**Incomes (baht/month)**							
Lower than 10,000	56	38	94	2.25	1.19 - 4.28	7.21	0.01*
10,000 and above	34	52	86				
Total	90	90	180				
							
**Having a physical illness**							
Presence	43	28	71	2.03	1.06 - 3.90	5.23	0.02*
Absence	47	62	109				
Total	90	90	180				

*p < 0.05, **p < 0.01

The multivariable analysis showed that the remaining factors associated with depressive disorder were four interpersonal problem areas: grief (adjusted OR = 6.01, 95%CI = 1.93 - 18.69, p < 0.01), interpersonal role disputes (adjusted OR = 6.01, 95%CI = 2.18 - 16.52, p < 0.01), role transitions (adjusted OR = 26.30, 95%CI = 7.84 - 88.25, p < 0.01), and interpersonal deficits (adjusted OR = 2.92, 95%CI = 1.12 - 7.60, p < 0.05). The sociodemographic factors (low education, unemployment, and having a physical illness) were not found to be associated with depressive disorder (Table 4).

**Table 4:** Multivariable analysis of factors associated to depressive disorder in Thai depressed patients

Variables	Coefficient (β)	p-value	Adjusted odds ratio (OR)	95% CI of adjusted OR
**Interpersonal problem areas**				
- Grief	1.793	0.002**	6.01	1.93 - 18.69
- Interpersonal role disputes	1.793	0.001**	6.01	2.18 - 16.52
- Role transitions	3.270	< 0.001**	26.30	7.84 - 88.25
- Interpersonal deficits	1.070	0.029*	2.92	1.12 - 7.60
**Sociodemographic factors**				
- Low education	0.775	0.117	2.17	0.82 - 5.72
- Unemployment	0.786	0.112	2.19	0.83 - 5.78
- Having a physical illness	-0.507	0.346	0.60	0.21 - 1.73

*p < 0.05, **p < 0.01

## Discussion

Most of the subjects in this study were educated and employed, young to middle-aged women living in Bangkok and the Central Provinces. The factors associated with depressive disorder in Thai depressed patients were all four interpersonal problem areas: grief, interpersonal role disputes, role transitions, and interpersonal deficits (p < 0.01); and certain sociodemographic factors: low education, unemployment, low income, and having a physical illness (p < 0.05). After performing a multivariable analysis, only the four interpersonal problem areas: grief, interpersonal role disputes, role transitions (p < 0.01), and interpersonal deficits (p < 0.05) remained. This indicates that the problem areas are more closely associated with depressive disorder than the sociodemographic variables.

Among interpersonal problem areas, role transitions had the strongest association with depressive disorder (adjusted OR = 26.30, 95% CI = 7.84 - 88.25, p < 0.01). The subjects in this study were in young to middle-aged adulthood; therefore, life changes were important issues in this stage [26]. Many people reported unsatisfactory experiences when having to adjust to major life changes such as separation or divorce, job loss, physical illness, and financial problems. Difficulties in adjusting to a new role may be due to loss of social support from the old role, feeling uncomfortable with the new role, or perceiving the new role as overwhelming or anxiety-provoking [8]. Previous research determined that widowhood promotes anxiety and depression by increasing concerns about living alone and loneliness [27], job loss heightened a two- to three- fold rate of anxiety and depression by increasing financial strain and heightening reactivity to stress [27,28]. Previous studies in Thai depressed patients showed that the adverse life events associated with depression were major health problems, financial problems, job loss, separation or divorce, and being unable to adjust to life change [29,30].

Grief or complicated bereavement, especially spousal bereavement, is the most stressful life event precipitating depression [18,19]. One study determined that annually in the US, approximately 800,000 people were newly widowed and bereaved [31]. Bereavement was found to lead to chronic depression in approximately 10 - 15% of cases and depressive disorder was found in 24 - 42% of the bereaved at 1 month, 16% at 1 year, but was found in only 8% of the non-bereaved [32-35]. In a previous study in Thailand, the death of a loved one was associated with depression as well [29]. In the present study, grief was found as an interpersonal problem area related to depression, but this problem area did not show the highest strength of association among other problem areas (adjusted OR = 6.01, 95%CI = 1.93 - 18.69, p < 0.01). This may be due to the relatively young age of the samples.

Interpersonal role disputes is another interpersonal problem area associated with depressive disorder in this study (adjusted OR = 6.01, 95%CI = 2.18 - 16.52, p < 0.01). Interpersonal role disputes include arguments or disagreements with a spouse (marital conflicts), family member, boss, colleague or co-worker, or a close friend [7-9]. Although interpersonal disputes are common, they may become a problem when they can not be resolved or remain chronic [8], leading to frustration, anger, and despair. Depressed patients with disputes tend to have maladaptive communication patterns such as ambiguous or indirect verbal and nonverbal communication, low assertiveness, an incorrect assumption that others understood their opinions or their needs, or closing off communication or being silent [7-9]. Previous studies showed that depressed patients had more problematic interpersonal relationships with their spouses and families than the non-depressed individuals [36-39]. Regarding the quality of interaction, the depressed individuals had significantly fewer positive interactions and more negative interactions with their spouses or partners than non-depressed ones [40]. A study in Thailand found depressed women to have significantly higher interpersonal conflicts than non-depressed women [41].

Interpersonal deficits were also found as an interpersonal problem area related to depressive disorder, but in the weakest association (adjusted OR = 2.92, 95%CI = 1.12 - 7.60, p < 0.05). Interpersonal deficits include lack of interpersonal or social skills and lack of social support [7-9]. Some indicators for interpersonal deficits include limited friends or family contact, lack of socially rewarding relationships, and repeated relationship failures [8]. People with interpersonal deficits usually have difficulty in life adjustment when experiencing interpersonal crises such as grief, or role transitions because they have difficulty in developing social connections with others after life changes [8]. Previous studies confirmed that poor social support was related to the onset, relapse, and recurrence of depressive disorder [36]. In Thailand, poor social support was associated with the depressive disorder in Thai women [41]. In the present study, interpersonal deficits were shown to have the weakest association with depressive disorder in Thai depressed patients. This may relate to the Thai socio-cultural system and Thai family structure. Thai people, as compared to Westerners, have large extended families and close connections to their families and relatives. The results of the present study suggest that interpersonal deficits are less relevant and can be disregarded as an IPT focus in Thailand. IPT was first developed in the treatment of white middle-class women in the Boston area of the United States of America. The present study addresses the universality and applicability of IPT in Thailand. In Thailand, people's character and culture differ from those of Western countries. Thais' manners and culture extend mainly from farming and Buddhism. The lifestyle of Thais is simple, easy, and generous. Thai people like to live together in cooperation and tend to have large extended families composed of grandparents, parents, sons or daughters, and grandchildren. In this culture, younger generations are taught to respect their elders and to be grateful to their parents and older relatives by taking care of them. However, compared to Westerners, Thais are more dependent and may be less assertive. When aiming to improve communication in Thai depressed patients, IPT therapists should work within the framework of the Thai lifestyle and culture.

As discussed above, although the socio-cultural context in Thailand is different from the West, the same interpersonal difficulties are endorsed by Thai depressed patients, but vary in degree.

This study attempted to reduce confounding factors by using the same-based controls from the hospital. However, the findings should be interpreted in the context of depressed patients in a clinical setting. These factors may have influenced the interpersonal problem areas that they experienced. In addition, this study is an analytic or case-control study trying to identify the interpersonal or social risks of depressive disorder in Thai depressed patients. However, tracing back the history of experiencing interpersonal events over the past year may result in recall bias in the subjects. Further prospective or cohort studies may help to identify more causal effects of these risks on depressive disorder.

## Conclusion

The study of universality of interpersonal problem areas in Thai depressed patients showed that grief, interpersonal role disputes, role transitions, and interpersonal deficits were all increased in depressed subjects as compared to non-depressed subjects, with role transitions having the strongest association with depressive disorder and interpersonal deficits the weakest. This finding makes interpersonal psychotherapy, which deals with these interpersonal difficulties, a suitable treatment for Thai depressed patients.

## Competing interests

Dr. Lueboonthavatchai and Dr. Thavichachart are both affiliated with the Department of Psychiatry, Faculty of Medicine, Chulalongkorn University, Rama 4 Road, Patumwan District, Bangkok 10330, Thailand. The authors both declare that they have no financial or non-financial competing interests.

## Authors' contributions

PL was the principal investigator for the study (conception and design of the study, literature review, protocol preparation, conducting the study, data collection, data analysis, interpretation of the results, and manuscript preparation and revision). NT contributed to the conception and design, interpretation of the results, revision and approval of the manuscript.

## Pre-publication history

The pre-publication history for this paper can be accessed here:

http://www.biomedcentral.com/1471-244X/10/87/prepub
